# A nomogram for predicting cancer-specific survival and overall survival in elderly patients with nonmetastatic renal cell carcinoma

**DOI:** 10.3389/fsurg.2022.1018579

**Published:** 2023-01-06

**Authors:** Chenghao Zhanghuang, Jinkui Wang, Zhaoxia Zhang, Zhigang Yao, Fengming Ji, Li Li, Yucheng Xie, Zhen Yang, Haoyu Tang, Kun Zhang, Chengchuang Wu, Bing Yan

**Affiliations:** ^1^ Department of Urology, Kunming Children’s Hospital, Children’s Hospital Affiliated to Kunming Medical University, Yunnan Province Clinical Research Center for Children’s Health and Disease, Kunming, China; ^2^ Department of Urology, Chongqing Key Laboratory of Children Urogenital Development and Tissue Engineering, Chongqing Key Laboratory of Pediatrics, Ministry of Education Key Laboratory of Child Development and Disorders, National Clinical Research Center for Child Health and Disorders, China International Science and Technology Cooperation Base of Child Development and Critical Disorders, Children’s Hospital of Chongqing Medical University, Chongqing, China; ^3^ Yunnan Key Laboratory of Children’s Major Disease Research, Kunming Children’s Hospital, Children’s Hospital Affiliated to Kunming Medical University, Kunming, China; ^4^ Department of Pathology, Kunming Children's Hospital, Children’s Hospital Affiliated to Kunming Medical University, Kunming, China; ^5^ Department of Oncology, Yunnan Children Solid Tumor Treatment Center, Kunming Children’s Hospital, Children’s Hospital Affiliated to Kunming Medical University, Kunming, China

**Keywords:** nomogram, cancer-specific survival, elderly patients, nonmetastatic renal cell carcinoma, SEER, overall survival

## Abstract

**Background:**

Renal cell carcinoma (RCC) is a common malignant tumor in the elderly, with an increasing trend in recent years. We aimed to construct a nomogram of cancer-specific survival (CSS) and overall survival (OS) in elderly patients with nonmetastatic renal cell carcinoma (nmRCC).

**Methods:**

Clinicopathological information was downloaded from the Surveillance, Epidemiology, and End Results (SEER) program in elderly patients with nmRCC from 2010 to 2015. All patients were randomly assigned to a training cohort (70%) or a validation cohort (30%). Univariate and multivariate Cox regression analyses were used to identify independent risk factors for patient outcomes in the training cohort. A nomogram was constructed based on these independent risk factors to predict the 1-, 3-, and 5-year CSS and OS in elderly patients with nmRCC. We used a range of methods to validate the accuracy and reliability of the model, including the calibration curve, consistency index (C-index), and the area under the receiver operating curve (AUC). Decision curve analysis (DCA) was used to test the clinical utility of the model.

**Results:**

A total of 12,116 patients were enrolled in the study. Patients were randomly assigned to the training cohort (*N* = 8,514) and validation cohort (*N* = 3,602). In the training cohort, univariate and multivariate Cox regression analysis showed that age, marriage, tumor histological type, histological tumor grade, TN stage, tumor size, and surgery are independent risk factors for prognosis. A nomogram was constructed based on independent risk factors to predict CSS and OS at 1-, 3-, and 5- years in elderly patients with nmRCC. The C-index of the training and validation cohorts in CSS were 0.826 and 0.831; in OS, they were 0.733 and 0.734, respectively. The AUC results of the training and validation cohort were similar to the C-index. The calibration curve indicated that the observed value is highly consistent with the predicted value, meaning the model has good accuracy. DCA results suggest that the clinical significance of the nomogram is better than that of traditional TNM staging.

**Conclusions:**

We built a nomogram prediction model to predict the 1-, 3- and 5-year CSS and OS of elderly nmRCC patients. This model has good accuracy and discrimination and can help doctors and patients make clinical decisions and active monitoring.

## Introduction

Renal cell carcinoma (RCC) is adults' most common renal malignant tumor, accounting for about 2%–3% of systemic neoplastic diseases and 90% of Renal tumors (1). Around the world, 400,000 people have diagnosed with RCC annually (2), and the elderly over 60 years old account for more than 75% of the cases (3). In addition, with the aggravation of population aging and extending life expectancy, the incidence rate of renal cancer in the elderly is also increasing yearly (1). Renal cell carcinoma is classified as either metastatic renal cell carcinoma (mRCC) or nonmetastatic renal cell carcinoma (nmRCC), depending on whether the tumor has metastasized. mRCC accounts for about 20% of the total RCC at the diagnosis, with a poor prognosis and median survival time of only 24 months (4). NmRCC is considered to have the potential of a complete cure (5). The prediction of elderly nmRCC varies significantly due to comorbidity and other factors (1). Therefore, an accurate prediction model is crucial in making a clinical decision, building patient confidence, and improving medical reliability.

The nomogram has been widely used to predict the survival of various cancers. Cui et al. (6, 7) predicted the recurrence and survival risk of patients with laryngeal squamous cell carcinoma using the nomogram.

Studies have shown that clinicopathological factors such as gender, tumor side, tumor size, and surgical approach significantly correlate with the prognosis of elderly patients with RCC (8–10). Wang et al. found that race, tumor histological type, histological grade, *T* stage, *N* stage, tumor size, surgery, radiotherapy, and chemotherapy were independent risk factors for distant metastasis of elderly RCC (35,155,365) (11). Guo et al. found that right renal cancer was an independent prognostic factor for cancer-specific survival in the subgroup of RCC with tumor size ≥10 cm (31,407,495) (12). Aron et al. concluded through significant sample analysis that men with larger, higher stage, and higher grade RCC have a higher overall survival rate than women and women (18,160,207) (13). Chen et al. found that partial nephrectomy for elderly patients with RCC who met specific clinical characteristics (such as tumor size ≤7 cm, N0 stage, or isolated metastasis) seemed to help improve survival prognosis (32,256,058) (14).

However, no specific prediction model for elderly patients with nmRCC has been reported. We developed a nomogram prediction model and validated its accuracy in evaluating cancer-specific survival rates, providing a reference for clinical diagnosis and treatment based on the above situation.

## Methods

### Dataset description

We downloaded the clinicopathological information of all elderly patients with nmRCC from 2010 to 2015 from the National Cancer Institute's Surveillance, Epidemiology, and Final Results (SEER) project. The SEER database is a public cancer database that includes 18 cancer registries in the United States, covering approximately 28% of the United States population (15). The SEER database can download patient demographic information, cancer characteristics, survival status, and other information. Log in to http://seer.cancer.gov/ to get al.l the data of this study. Because the patient's lead in the SEER database is publicly available and does not contain identifiable personal data, this study does not require ethical approval and informed consent. Moreover, our research methods comply with the research rules of the SEER database.

### Preprocessing

We collected patient demographic information (age, sex, race, marital status, year of diagnosis), tumor characteristics (tumor laterality, tumor size, histopathological type, histopathological grade, *T* stage, *N* stage), and treatment (surgery, radiotherapy, chemotherapy). Inclusion criteria: (1) Age ≥65 years; (2) Pathological diagnosis of renal cell carcinoma (SEER database ICD-O-3 codes 8,260, 8,310, 8,312, 8,317); (3) The diagnosis year 2010–2015; (4) M staging is M0.

**Figure 1:**
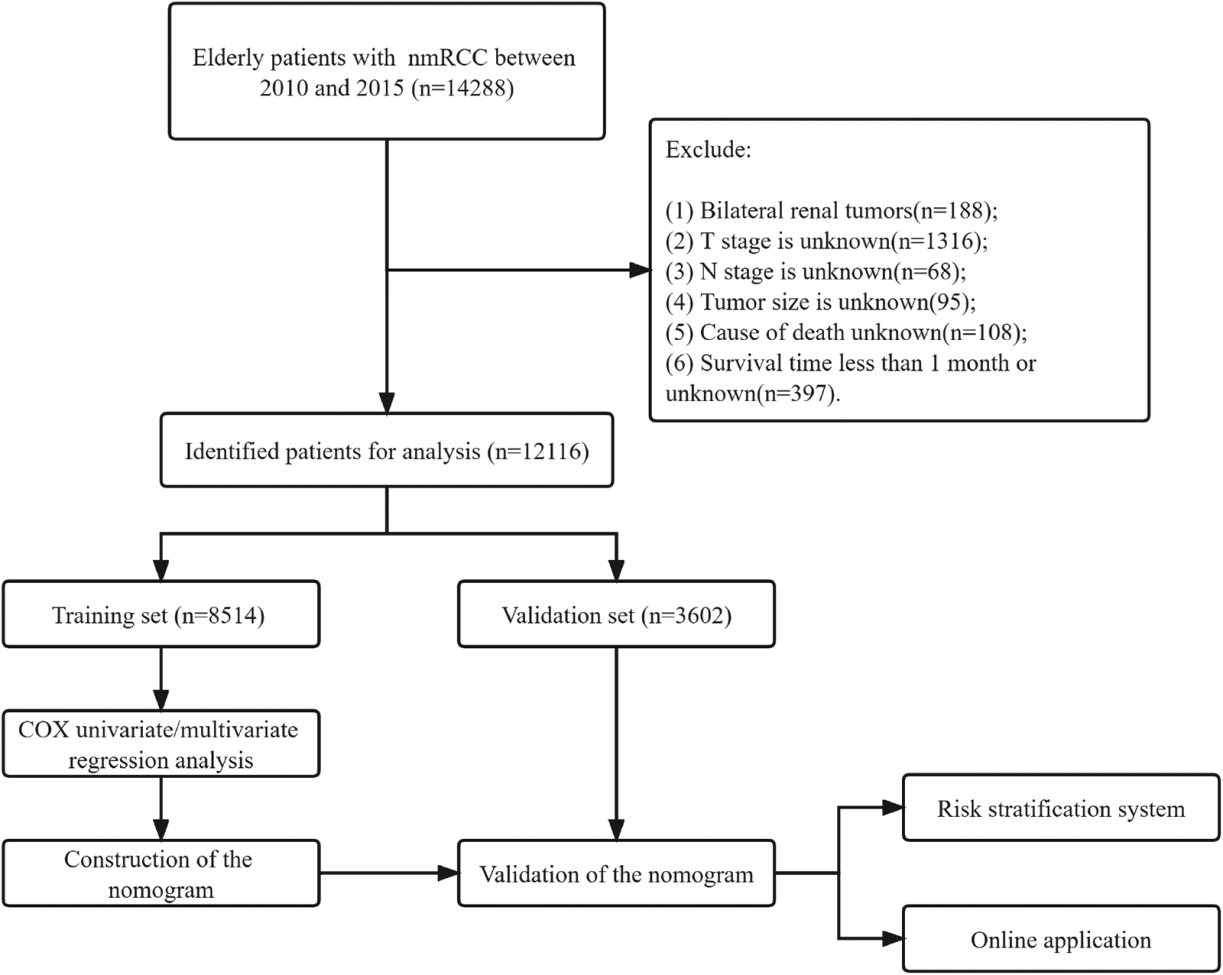
Flow chart for inclusion and exclusion of elderly patients with nmRCC. Patients in the training set were used to screen independent risk factors and establish a nomogram. The model's accuracy was checked using the validation set and the external validation set. Finally, a risk stratification system was established to identify high-risk patients, and the webpage was used to visualize the clinical application.

Exclusion criteria: (1) bilateral renal tumors; (2) *T* and *N* stages are unknown; (3) tumor size is unknown; (4) cause of death is unknown; (5) survival time is less than one month. The patient screening process is shown in Figure 1.

### Classification

The racial information of the patients was divided into white, black, and other (American Indian/AK Native, Asian/Pacific Islander). The histological grades of tumors were divided into grades I–IV, which are well-differentiated, moderately differentiated, poorly differentiated, and undifferentiated, and the histological grade of some patients is unknown. Histopathological types included renal clear cell carcinoma, papillary renal cell carcinoma, chromophobe cell carcinoma, and some unclassified renal cell carcinoma. According to the operation code, the patient's operation type was divided into local tumor excision (SEER operation code 10–27), partial nephrectomy (SEER operation code 30), and radical nephrectomy (SEER operation code 40–80).

### Nomogram construction and validation

All patients were randomly divided into a training cohort (70%) and a validation cohort (30%). In the training cohort, univariate and multivariate Cox regression model analyses were used to screen independent risk factors for CSS and OS of patients. The hazard ratio (HR) and 95% confidence interval (CI) were recorded. A new nomogram was developed to predict patients' 1-, 3-, and 5-year CSS and OS based on independent risk factors. The calibration curve of 1,000 bootstrap samples was used for internal validation to evaluate the model's accuracy. We used the consistency index (C-index) to assess the discriminative power of the predictive model. We also used the area under the receiver operating curve(AUC) of the model to evaluate the discriminatory ability of the model.

### Clinical utility

Decision curve analysis (DCA) is a new algorithm that evaluates the effectiveness of models based on the net income under different risk thresholds (16). We used DCA to analyze the clinical application value of the nomogram and compared it with traditional TNM staging. In addition, we divided all patients into high-risk and low-risk groups based on their nomogram scores. We used the log-rank test and Kaplan-Meier curve (K-M) to compare the survival differences of patients in different groups.

### Statistical analysis

All count data were described by frequency (%), and differences between groups were analyzed by *χ*^2^ and non-parametric *U* tests. We described measurement data (tumor size, age) using mean and standard deviation, and differences between groups were analyzed using a non-parametric *U* test. The Cox regression model analyzed independent risk factors for patient survival and prognosis. Differences in survival between groups were analyzed using the Kaplan-Meier curve and log-rank test. All statistical analysis uses SPSS 26.0 and *R* software 4.1.0. A P value of less than 0.05 is considered statistically significant.

## Results

### Clinical features

A total of 12,116 patients were included in the study. The patients were randomly divided into a training cohort (*N* = 8,514) and a validation cohort (*N* = 3,602). The average age of the patients was 72.9 years old, of which 9,901 (81.7%) were white, and 7,053 (58.2%) were male. There were 7,129 (58.8%) married patients. There were 1,028 (8.48%), 4,784 (39.5%), 2,831 (23.4%), and 533 (4.40%) patients with histological tumor grades I–IV, respectively. There were 6,000 (49.5%) patients with tumors on the left. The histological types of tumors in 7,193 (59.4%), 1,657 (13.7%), and 693 (5.72%) patients were clear cell type, papillary type, and chromophobic type, respectively. There were 8,633 (71.3%) patients with T1 stage tumors and 11,882 (98.1%) patients with N0 stage tumors. The average tumor size was 49 mm. Surgical methods included local tumor resection in 916 cases (7.56%), partial resection in 3,518 cases (29.0%), and radical resection in 6,261 cases (51.7%). 201 (1.66%) patients received chemotherapy, and 54 (0.45%) patients received radiotherapy. The clinicopathological information of all patients is shown in Table 1. There was no significant difference between the training cohort and the validation cohort.

**Table 1:** Clinicopathological characteristics of elderly patients with nmRCC.

	All	Training cohort	validation cohort	
	*N *= 12,116	*N *= 8,514	*N* = 3,602	*p*
Age	72.9 (6.52)	73.0 (6.53)	72.9 (6.49)	0.385
Race				0.062
White	9,901 (81.7%)	6,953 (81.7%)	2,948 (81.8%)	
Black	1,356 (11.2%)	931 (10.9%)	425 (11.8%)	
Other	859 (7.09%)	630 (7.40%)	229 (6.36%)	
Sex				0.193
Male	7,053 (58.2%)	4,989 (58.6%)	2,064 (57.3%)	
Female	5,063 (41.8%)	3,525 (41.4%)	1,538 (42.7%)	
Marriage				0.631
No	4,987 (41.2%)	3,492 (41.0%)	1,495 (41.5%)	
Married	7,129 (58.8%)	5,022 (59.0%)	2,107 (58.5%)	
Histologic.type				0.857
Clear cell	7,193 (59.4%)	5,075 (59.6%)	2,118 (58.8%)	
Papillary	1,657 (13.7%)	1,154 (13.6%)	503 (14.0%)	
Chromophobe	693 (5.72%)	483 (5.67%)	210 (5.83%)	
Not classified	2,573 (21.2%)	1,802 (21.2%)	771 (21.4%)	
Laterality				0.161
Left	6,000 (49.5%)	4,252 (49.9%)	1,748 (48.5%)	
Right	6,116 (50.5%)	4,262 (50.1%)	1,854 (51.5%)	
Grade				0.837
I	1,028 (8.48%)	722 (8.48%)	306 (8.50%)	
II	4,784 (39.5%)	3,338 (39.2%)	1,446 (40.1%)	
III	2,831 (23.4%)	2,005 (23.5%)	826 (22.9%)	
IV	533 (4.40%)	370 (4.35%)	163 (4.53%)	
Unknown	2,940 (24.3%)	2,079 (24.4%)	861 (23.9%)	
T				0.998
T1	8,633 (71.3%)	6,068 (71.3%)	2,565 (71.2%)	
T2	1,134 (9.36%)	795 (9.34%)	339 (9.41%)	
T3	2,270 (18.7%)	1,596 (18.7%)	674 (18.7%)	
T4	79 (0.65%)	55 (0.65%)	24 (0.67%)	
N				0.151
N0	11,882 (98.1%)	8,360 (98.2%)	3,522 (97.8%)	
N1	234 (1.93%)	154 (1.81%)	80 (2.22%)	
Tumor size	49.0 (31.4)	49.2 (32.1)	48.6 (29.7)	0.320
Surgery				0.820
No	1,421 (11.7%)	1,013 (11.9%)	408 (11.3%)	
Local tumor excision	916 (7.56%)	646 (7.59%)	270 (7.50%)	
Partial nephrectomy	3,518 (29.0%)	2,471 (29.0%)	1,047 (29.1%)	
Radical nephrectomy	6,261 (51.7%)	4,384 (51.5%)	1,877 (52.1%)	
Chemotherapy				0.786
No/Unknown	11,915 (98.3%)	8,375 (98.4%)	3,540 (98.3%)	
Yes	201 (1.66%)	139 (1.63%)	62 (1.72%)	
Radiation				0.240
No/Unknown	12,062 (99.6%)	8,468 (99.5%)	3,594 (99.8%)	
Yes	54 (0.45%)	46 (0.54%)	8 (0.22%)	

### Univariate and multivariate Cox regression analysis

We evaluated the proportional risk assumption using Schoenfeld residuals and Kaplan-Meier curves. We performed univariate Cox regression analysis on all variables in the training cohort to screen out survival-related variables. Results for CSS showed that age, sex, marriage, tumor histological type, histological tumor grade, *T* stage, *N* stage, tumor size, and surgery was associated with survival prognosis. In the OS nomogram we constructed, we found that the factors included in the prediction and CSS were the same. It is worth noting the differences and scores of these factors. We had the predictive variables in a multivariate Cox regression analysis, which showed that these variables were independent risk factors for CSS (Table 2) and OS (Table 3) of patients.

**Table 2:** Univariate and multivariate analyses of CSS in training set.

	Univariate			Multivariate		
	HR	95% CI	*p*	HR	95% CI	*p*
Age	1.07	1.06–1.08	<0.001	1.045	1.035–1.054	<0.001
Sex						
Male	reference					
Female	0.87	0.77–0.98	0.023	0.832	0.729–0.95	0.007
Year of diagnosis	0.95	0.92–0.99	0.016			
Marriage						
No	reference					
Married	0.72	0.63–0.81	<0.001	0.843	0.738–0.962	0.011
Histologic type						
Clear cell	reference					
Papillary	0.82	0.692–0.972	0.022	0.909	0.739–1.118	0.367
Chromophobe	0.336	0.232–0.486	<0.001	0.353	0.225–0.553	<0.001
Not classified	1.768	1.579–1.98	<0.001	1.112	0.939–1.317	0.217
Grade						
I	reference					
II	1.567	1.155–2.127	0.004	1.544	1.081–2.204	0.017
III	3.902	2.89–5.267	<0.001	2.53	1.771–3.614	<0.001
IV	10.293	7.473–14.178	<0.001	4.378	2.974–6.443	<0.001
Unknown	4.143	3.068–5.593	<0.001	1.673	1.16–2.413	0.006
T						
T1	reference					
T2	2.777	2.383–3.237	<0.001	1.802	1.468–2.212	<0.001
T3	3.645	3.258–4.08	<0.001	2.553	2.169–3.004	<0.001
T4	15.598	11.661–20.863	<0.001	4.339	2.998–6.28	<0.001
N						
N0	reference					
N1	9.24	7.47–11.43	<0.001	3.179	2.53–3.996	<0.001
Surgery						
No	reference					
Local tumor excision	0.171	0.129–0.224	<0.001	0.266	0.184–0.383	<0.001
Partial nephrectomy	0.1	0.082–0.121	<0.001	0.171	0.129–0.226	<0.001
Radical nephrectomy	0.399	0.354–0.451	<0.001	0.312	0.25–0.389	<0.001
Tumor size	1.01	1.009–1.011	<0.001	1.005	1.004–1.006	<0.001

**Table 3:** Univariate and multivariate analyses of OS in training set.

	Univariate			Multivariate		
	HR	95% CI	*p*	HR	95% CI	*p*
Age	1.08	1.07–1.08	<0.001	1.053	1.046–1.059	<0.001
Sex						
Male	reference					
Female	0.87	0.81–0.95	0.001	0.758	0.695–0.827	<0.001
Year of diagnosis	0.96	0.93–0.98	0.001			
Race						
White	reference					
Black	1.14	1.01–1.29	0.03	1.306	1.154–1.479	<0.001
Other	0.78	0.66–0.93	0.005	0.843	0.712–0.998	0.048
Marriage						
No	reference					
Married	0.66	0.61–0.72	<0.001	0.783	0.719–0.854	<0.001
Histologic type						
Clear cell	reference					
Papillary	0.9	0.79–1.03	0.122	0.916	0.801–1.048	0.201
Chromophobe	0.52	0.41–0.66	<0.001	0.543	0.427–0.69	<0.001
Not classified	1.93	1.77–2.1	<0.001	1.216	1.091–1.356	<0.001
Grade						
I	reference					
II	0.95	0.81–1.13	0.586	0.993	0.838–1.176	0.933
III	1.37	1.15–1.62	<0.001	1.189	0.995–1.419	0.056
IV	2.93	2.37–3.61	<0.001	1.862	1.493–2.322	<0.001
Unknown	2	1.7–2.36	<0.001	1.037	0.866–1.241	0.695
T						
T1	reference					
T2	1.57	1.38–1.79	<0.001	1.082	0.931–1.256	0.304
T3	1.86	1.69–2.03	<0.001	1.484	1.326–1.659	<0.001
T4	5.14	3.72–7.09	<0.001	1.938	1.387–2.708	<0.001
N						
N0	reference					
N1	4.79	3.97–5.78	<0.001	2.124	1.723–2.618	<0.001
Surgery						
No	reference					
Local tumor excision	0.25	0.21–0.29	<0.001	0.41	0.34–0.496	<0.001
Partial nephrectomy	0.14	0.12–0.16	<0.001	0.268	0.227–0.318	<0.001
Radical nephrectomy	0.34	0.31–0.37	<0.001	0.43	0.372–0.496	<0.001
Tumor size	1.01	1.008–1.012	<0.001	1.004	1.003–1.005	<0.001

### Nomogram construction

**Figure 2:**
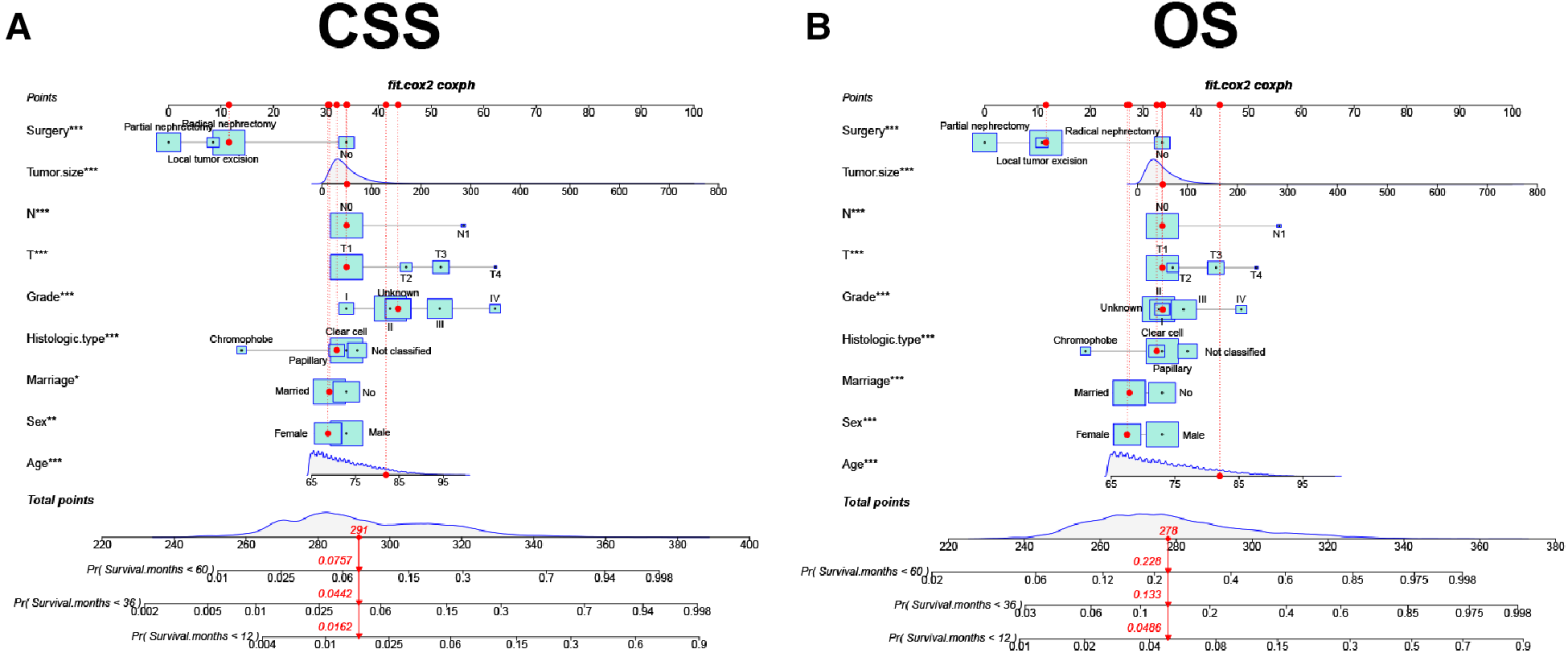
The nomogram of CSS and OS in elderly patients with nmRCC at 1-, 3-, and 5-year;.

A new nomogram was developed based on screened independent risk factors to predict CSS and OS in elderly patients with nmRCC (Figure 2). The nomogram showed that tumor size is the most significant factor influencing patient prognosis, both in CSS and OS. Followed by surgical method, age, *T* stage, histological tumor grade, tumor histological type, and *N* stage. Finally, marriage and sex have a minor influence on CSS.

### Validation

**Figure 3:**
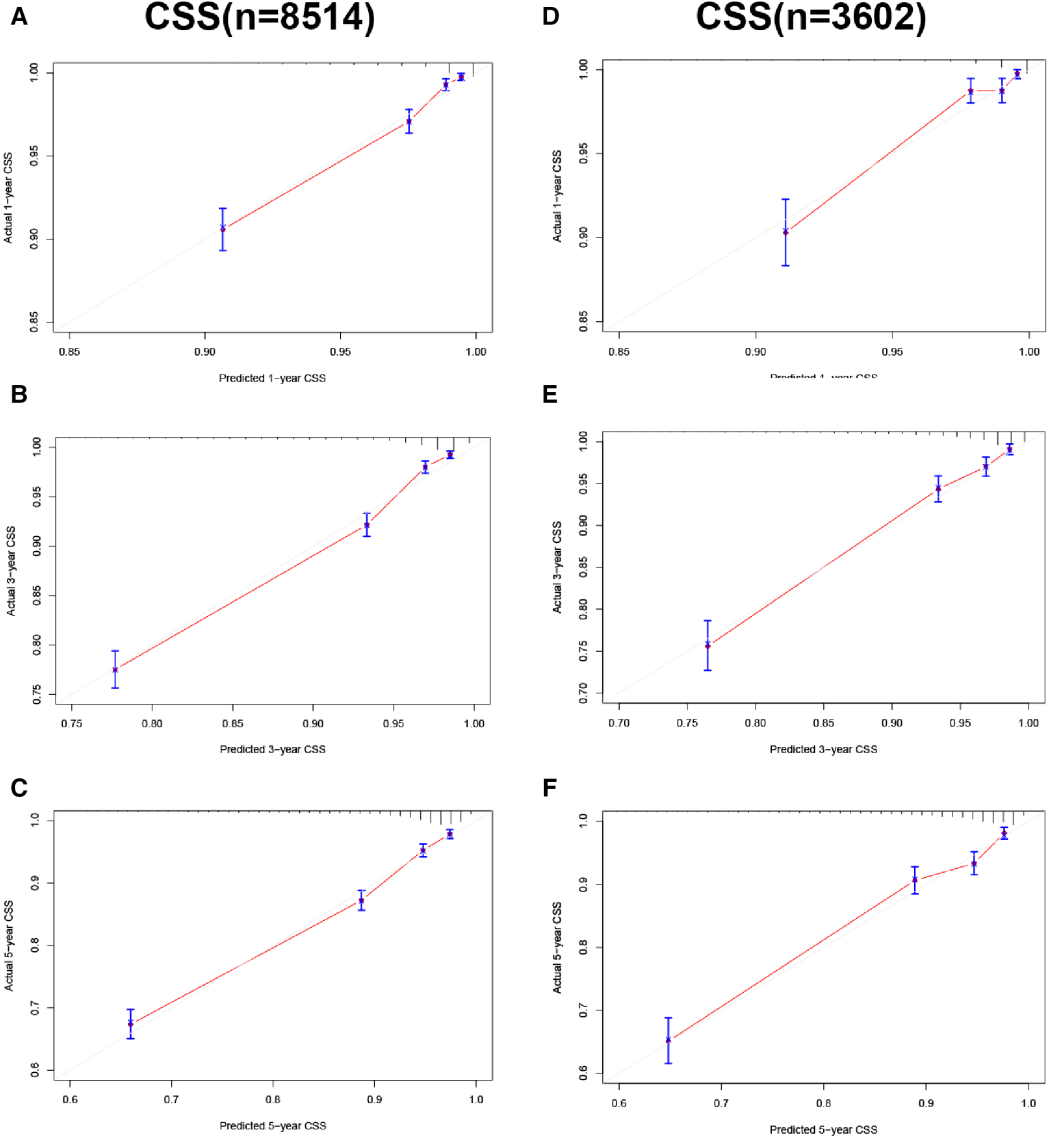
Calibration curve of the nomogram. (**A–C**) Calibration curves of 1-, 3- and 5-year CSS in the training cohort; (**D–F**) calibration curves of 1-, 3-, and 5-year CSS in the validation cohort.

**Figure 4:**
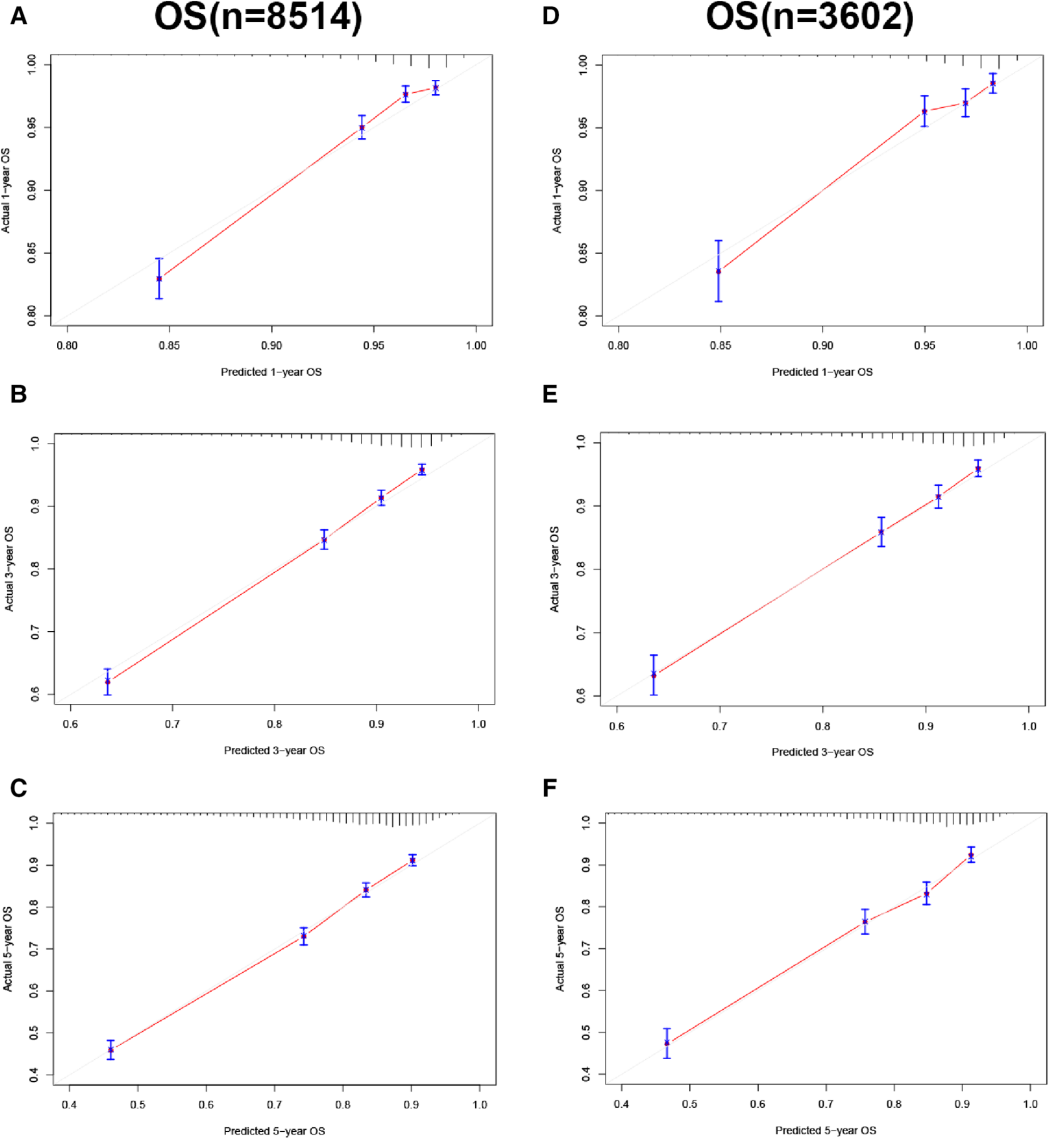
Calibration curve of the nomogram. (**A–C**) Calibration curves of 1-, 3- and 5-year OS in the training cohort; (**D–F**) calibration curves of 1-, 3-, and 5-year OS in the validation cohort.

**Figure 5:**
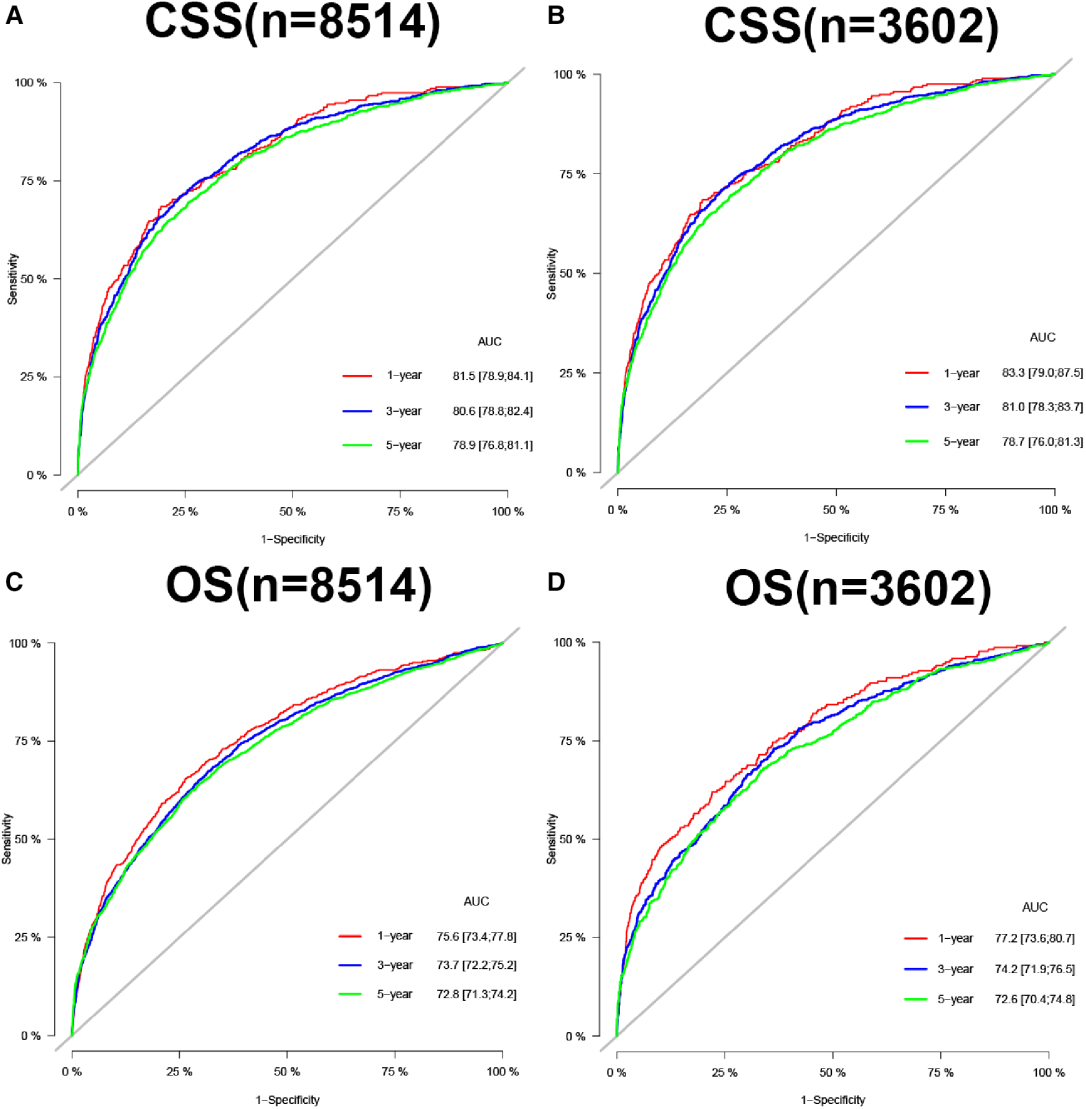
AUC for predicting 1-, 3-, and 5-year CSS in training cohort (**A**) and validation cohort (**B**), OS in training cohort (**C**), and validation cohort (**D**).

In the training and validation cohorts, the calibration curve suggested that the observed value was almost identical to the predicted value on CSS, proving our prediction model's excellent accuracy (Figures 3A–F). OS obtained similar results (Figures 4A–F). The training and validation cohort's C-index for CSS was 0.826 (95% CI: 0.814–0.838) and 0.831 (95% CI: 0.811–0.851), respectively. In OS, the C-index were 0.733 (0.723–0.743) and 0.734 (0.718–0.750), respectively. It shows that the model had good discrimination. In the CSS training cohort, the AUC of 1-, 3- and 5-year were 0.815, 0. 806, and 0.789, respectively. In the CSS validation cohort, the AUC values of 1-, 3- and 5-year were 0.833, 0.810, and 0.787, respectively. Similarly, In the OS training cohort, the AUC of 1-, 3- and 5-year were 0.756, 0. 737, and 0.728, respectively. In the OS validation cohort, the AUC values of 1-, 3- and 5-year were 0.772, 0.742, and 0.726, respectively. The result showed that the nomogram has good discrimination (Figure 5).

### Clinical application

**Figure 6:**
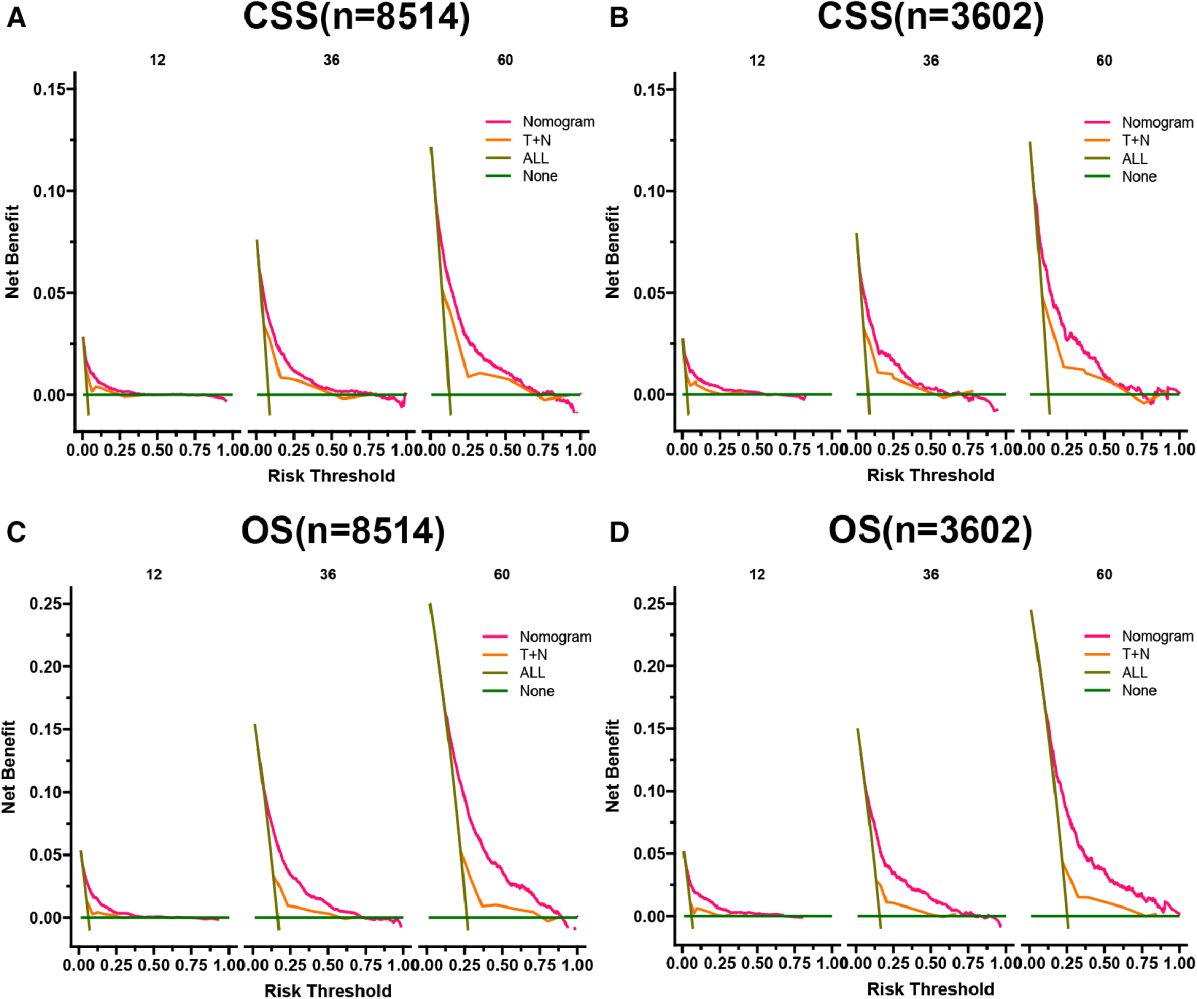
DCA of the CSS nomogram in training cohort (**A**) and validation cohort (**B**), OS nomogram in training cohort (**C**), and validation cohort (**D**). The *y*-axis represents a net benefit, and the *x*-axis represents threshold probability. The green line means no patients died, and the dark green line means all patients died. When the threshold probability is between 0% and 75%, the net benefit of the model exceeds all deaths or none.

In CSS and OS nomograms, both the training and validation cohorts, the DCA of the nomogram has a better clinical application value than the traditional TNM staging (Figure 6). In addition, we developed a risk stratification system in which patients were divided into a high-risk group (overall score >78.3) and a low-risk group (overall score ≤78.3) for CSS. K-M curve showed that the 1-, 3-, and 5-year survival rates were 92.3%, 81.6%, and 73.3% in the high-risk group, and 99.2%, 97.7%, and 95.1% in the low-risk group. Similarly, in terms of OS, elderly nmRCC patients included in the study were divided into the high-risk group (overall score >64.2) and the low-risk group (overall score ≤64.2) by the optimal cut-off value method. The 1-, 3- and 5-year survival rates of the high-risk group were 90.1%, 74.9%, and 61.7%, respectively. The survival rates in the low-risk group were 98.2%, 94.2%, and 88.5%, respectively.

**Figure 7:**
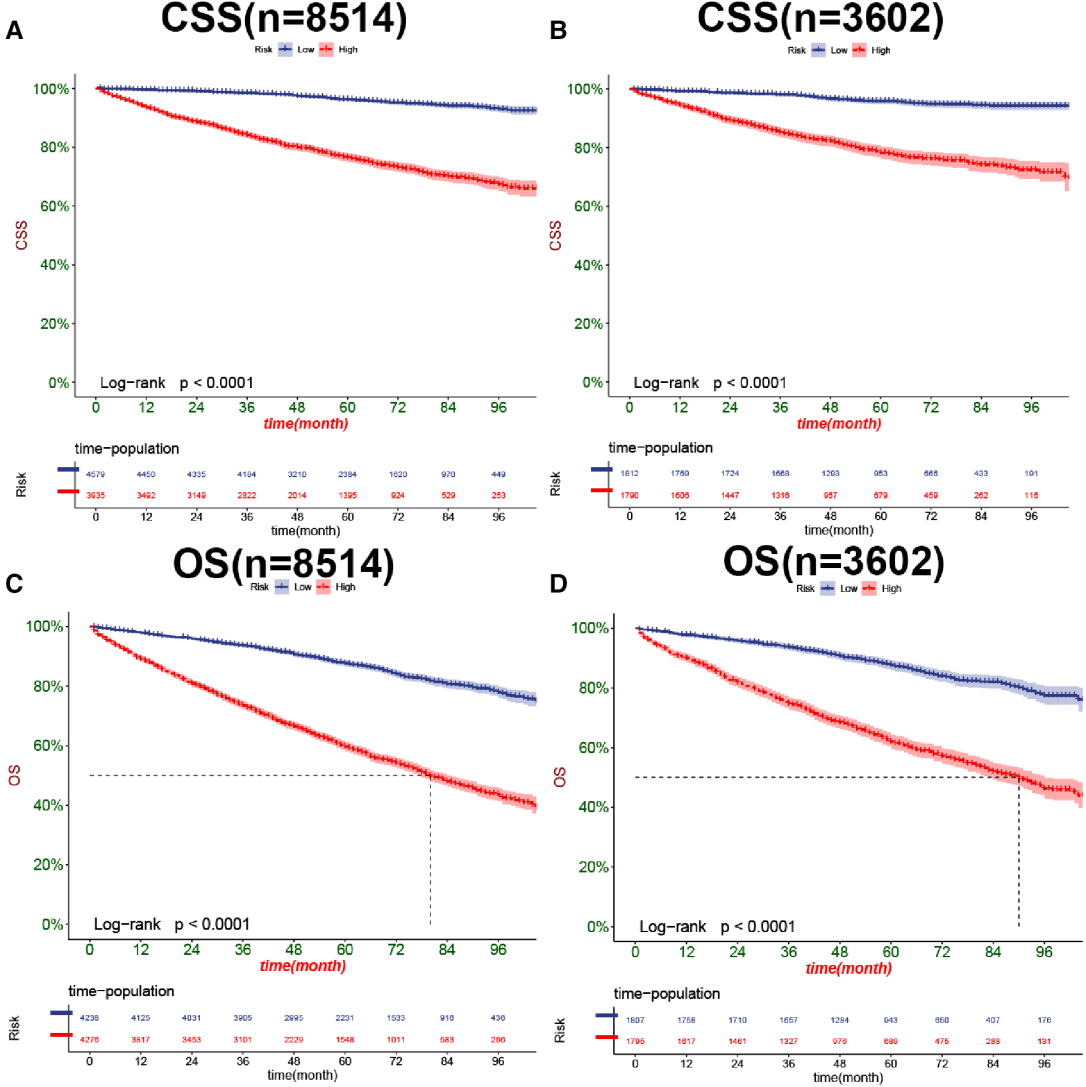
Kaplan-Meier curves of patients for CSS in the low-risk and high-risk groups in training cohort (**A**) and validation cohort (**B**), OS in the low-risk and high-risk groups in training cohort (**C**), and validation cohort (**D**).

**Figure 8:**
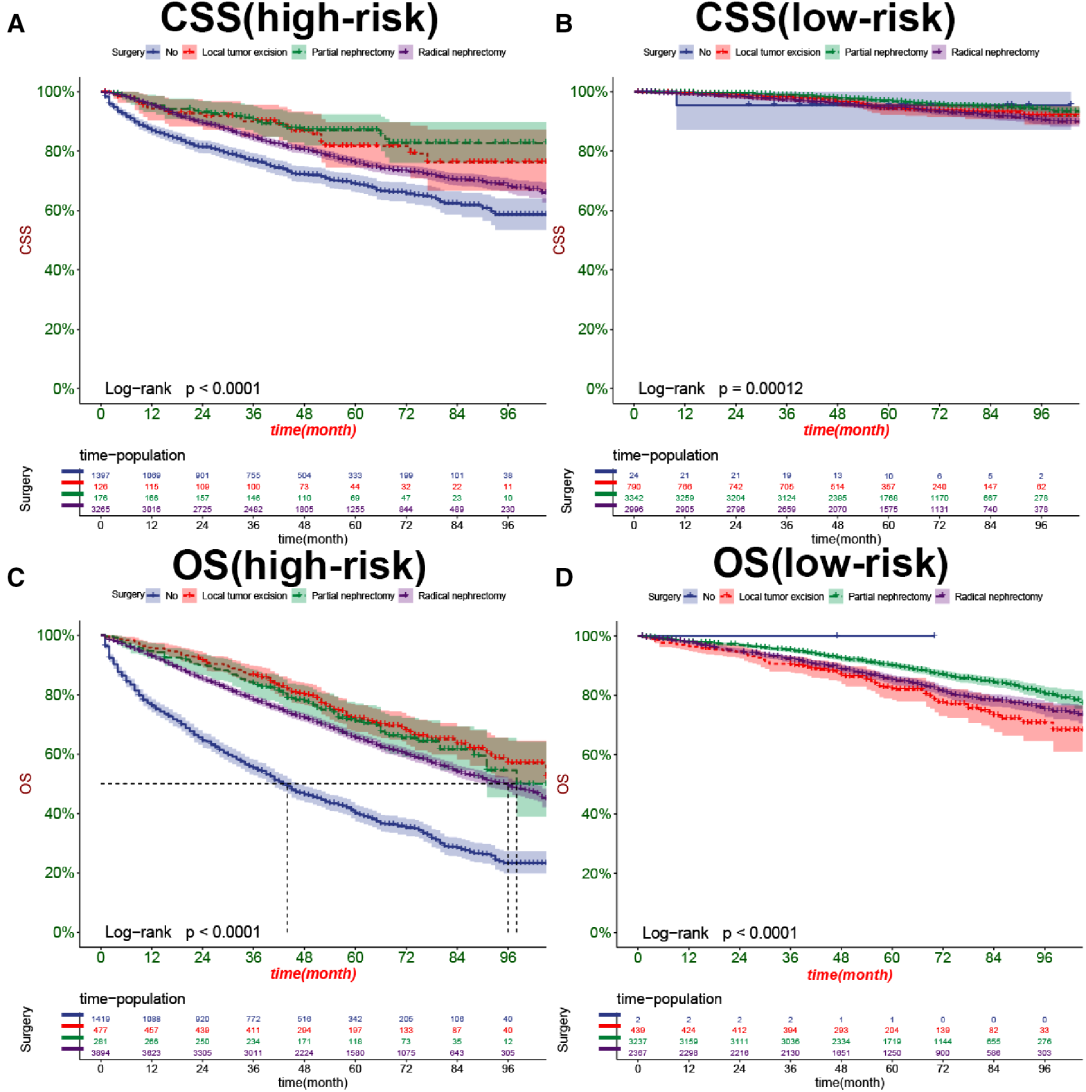
Kaplan-Meier curves of patients with different surgical procedures in the high-risk group (**A,C**) and low-risk group (**B,D**).

In both the training and validation cohorts, K-M curves showed that the survival rate of patients in the high-risk group was significantly lower than that in the low-risk group, either CSS (Figures 7A,B) or OS (Figures 7C,D). We also analyzed patients' surgical options based on risk stratification. Most patients underwent radical nephrectomy in the high-risk group, and survival was significantly higher than patients who did not undergo surgery. A few patients underwent local tumor excision and partial nephrectomy, and the survival rate was considerably higher than that of patients with radical resection, CSS (Figure 8A), and OS (Figure 8C). In the low-risk group, a small number of patients underwent local tumor excision. Most patients chose partial nephrectomy, radical nephrectomy, CSS (Figure 8B), and OS (Figure 8D).

### Online application

We developed a web application to predict elderly nmRCC patients based on the nomogram we constructed. Visit https://zhanghuang.shinyapps.io/DynNomapp/ to enter the site. The clinicopathological characteristics of the patient, such as age, gender, and tumor size, were input, and the “predict” button was clicked (Supplementary material Figure S1A). A patient's survival rate plot was displayed on the right side of the page (Supplementary material Figure S1B). In addition, the survival rate and confidence interval at a particular time point can be accurately calculated (Supplementary material Figure S1C), which provides an individualized and visual reference for the clinical treatment of elderly nmRCC patients.

## Discussion

Renal cell carcinoma is a type of cancer composed of different histopathological subtypes, mainly clear cell carcinoma, papillary cell carcinoma (types 1 and 2), and chromophobe cell carcinoma, with various genetic and molecular changes (17). Loss of heterozygosity (LOH) on chromosome 3 broken arm (3P) is common in kidney cancer and is considered to be a common feature of different subtypes of kidney cancer (18). The kidney is hidden behind the peritoneum, which is difficult to detect early. In addition, due to low immunity, some elderly patients may present with perirenal abscesses.

Age is a critical factor in cancer development, as is kidney cancer, where the risk of genetic mutations that trigger cancer increases with age. Cellular mutations are often considered the first step in the development of cancer. Aging is associated with highly reproducible DNA methylation changes, which helps explain the higher prevalence of malignant tumors in the elderly (19). Multiple studies have shown that patients aged ≥65 were defined as the elderly (20, 21). The current prediction model of renal cancer mainly targets patients of all ages but ignores the psychosocial and physiological changes of the elderly, such as reduced heart and lung compliance and bone fragility, which leads to activity limitation, thrombosis, and various comorbidities such as hypertension (22).

Given a large number of comorbid conditions in older adults, non-cancer-specific deaths (nCSD) often become nonnegligible. We performed nomogram construction and validation for both CSS and OS of elderly nmRCC in this study to ensure the rigor of our research.

In the current essential research stage, the genetic information of many diseases has been elucidated, and it has been proved that men and women are genetically distinct. However, gender differences are often ignored in clinical studies (23). On average, two individuals of the same sex are more than 99.9 percent alike. However, the genetic similarity between male and female individuals is only 98.5% (24). Genetic differences are an essential part of gender differences. Genetic differences are closely related to hereditary kidney cancer, so we should take gender differences into account in predicting the prognosis of the disease and evaluating the treatment effect (25). Kunath et al. (26) showed that gender is an independent risk factor for predicting renal cell carcinoma. At the same time, gender was closely related to pathological classification. Chromophobe cell carcinoma was predominant in female RCC patients, and papillary cell carcinoma was rare. The low aggressiveness of chromophobe cell carcinoma provides strong evidence that women with RCC have a better prognosis than men.

Many studies have shown that married patients have an increased relative-survival rate (RR) of cancer (27–29). Compared with single patients, married cancer patients can achieve early detection, timely operation, and maintain long-term survival (30). It may be related to the change in psychological state after marriage. John et al. (31) found that the incidence of depression in men after marriage was significantly lower than when they were single. Perini et al. (32) also found that depression was associated with lower survival and compliance with cancer. In conclusion, married patients showed significant improvement in RR for relatively low-grade cancers, such as the nmRCC included in this study. Marriage is not beneficial for the most aggressive forms of cancer, such as pancreatic cancer (33).

Historically, pathological classification of RCC mainly depended on morphology, but improved molecular and genetic studies will help better variety, treatment selection, and prognosis assessment. Combined with the latest consensus of the International Society of Urological Pathology (ISUP) (34), Clear cell renal cell carcinoma (CCRCC) is the most common subtype of renal cell carcinoma, accounting for 65%–70% of renal tumors (35), Carbonic anhydrase IX (Carbonic anhydrase IX), CAIX) and CD10 membrane positivity is considered to be a relatively specific diagnostic indicators. Papillary renal cell carcinoma (PRCC) is the second most common type of RCC, accounting for 15%–19% of adult renal carcinoma (36). The papilla is classified as type 1 and type 2. Type 1 papilla is covered with cuboidal cells and abnormalities on chromosome 7 or 17. We should carefully differentiate the histomorphologic variation of type 2 from other renal tumors. Chromophobe cell renal cell carcinoma (CRCC) accounts for 6%–11% of renal epithelial neoplasms. Microscopically, it has apparent morphological features, such as pale cells with clear cell boundaries and small eosinophilic neoplasms.

In this study, TNM staging of RCC was performed according to the updated criteria of the European Association of Urology (EAU) in 2021 (37), and PT1-4, N0-1, and M0 were used as the diagnostic criteria of nmRCC. Fuhrman grading system was adopted for tumor classification (38). According to WHO recommendations, grade I was defined as well-differentiated, grade II as moderately differentiated, grade III as poorly differentiated, and grade IV as undifferentiated.

Radical nephrectomy (RN) is the preferred treatment for renal cell carcinoma. However, there is increasing evidence that surgically induced chronic kidney disease can increase the mortality of patients (39). With the strengthening of awareness of prevention and health care and the development of imaging examinations, the early diagnosis rate of renal cancer has increased. Even the elderly are dominated mainly by localized and small focal tumors (1).

Therefore, many treatments for nephrectomy (PN) and radiofrequency ablation (RFA) are widely used in clinical practice (40). In elderly patients, the treatment of nmRCC carcinoma must consider surgical risk, comorbidity, and other competing causes of death, and 20% of renal masses are pathologically diagnosed as benign (41). Therefore, to avoid complications caused by surgical procedures, active surveillance (AS) has also begun to be used to treat clinical nmRCC.

In this study, we used the data of cancer patients in the SEER database to construct CSS and OS prediction models for elderly patients with nmRCC. Internal validation of the prediction model in the training and validation cohorts showed that the model had a high C-index of 0.826 (95% CI: 0.814–0.838) and 0.831 (95% CI: 0.811–0.851) in CSS. At the same time 0.733(95% CI: 0.723–0.743)and 0.734(95% CI: 0.718–0.750) in OS. Which proved that it had good accuracy. DCA of the training and validation cohorts also suggested that CSS and OS prediction model has better clinical application value than the traditional TNM staging system and can more accurately predict the survival prognosis of elderly patients with nmRCC.

There are some limitations to our study. First of all, our study is a retrospective study, which inevitably has a selection bias that is difficult to adjust. For example, patients in the high-risk group who underwent local tumor excision and PN may have a higher survival rate because of the lower tumor stage. Secondly, our study did not include factors that may affect prognoses, such as obesity, smoking, drinking, and hypertension, so that the results may be biased to some extent. However, we included critical prognostic factors such as tumor stage, size, surgery, and age so that the results would be unbiased. Finally, our prediction model has only been validated internally, and external validation or further prospective studies are necessary.

## Conclusion

This study explored the prognostic factors of CSS and OS in elderly patients with nmRCC and found that age, gender, marriage, tumor histological type, histological tumor grade, *T* stage, *N* stage, tumor size, and surgery were independent risk factors for patient prognosis. In addition, we built a nomogram prediction model to predict 1-, 3- and 5-year of CSS and OS for elderly patients with nmRCC. This model has good accuracy and discrimination and can provide a basis for doctors to make individualized clinical decisions most suitable for elderly patients with nmRCC.

## Data Availability

Publicly available datasets were analyzed in this study. This data can be found here: https://seer.cancer.gov/.
