# Supplementary material for: A nomogram for predicting cancer-specific survival and overall survival in elderly patients with nonmetastatic renal cell carcinoma
Source: Front Surg. 2023 Jan 6;9:1018579. doi: 10.3389/fsurg.2022.1018579 (PMC9852727; doi:10.3389/fsurg.2022.1018579)
Supplement: Supplementary file 1 [file Image1.docx]

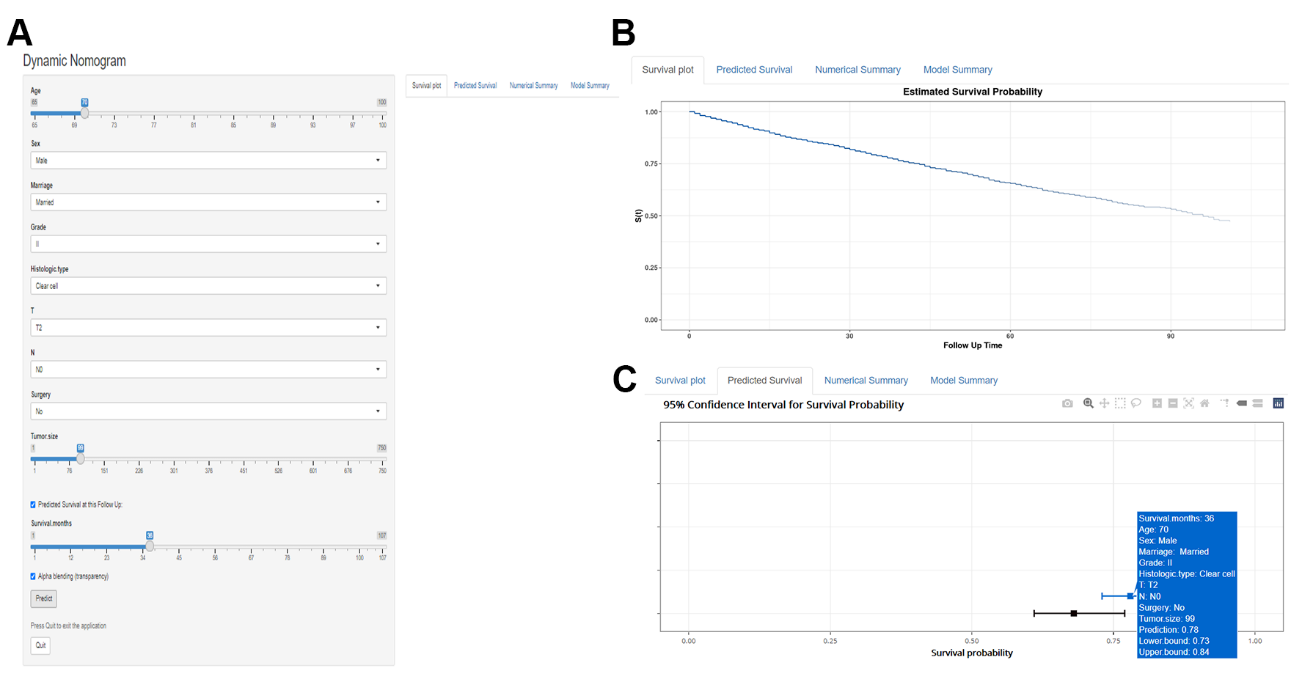


**Figure S1.** Dynamic nomogram in the URL landing page (A). The relevant clinicopathological factors of nmRCC patients are entered, and the plot of patient survival is shown on the right side (B). Accurate presentation of survival rates and 95% confidence intervals for this nmRCC patient(C).
